# Budget consolidation in a small open economy: a case study for Slovenia

**DOI:** 10.1080/14631377.2018.1537735

**Published:** 2019-01-11

**Authors:** Dmitri Blueschke, Klaus Weyerstrass, Reinhard Neck, Boris Majcen, Andrej Srakar, Miroslav Verbič

**Affiliations:** a Department of Economics, Alpen-Adria-Universität Klagenfurt, Klagenfurt, Austria; b Institute for Advanced Studies, Macroeconomics and Public Finance Group, Vienna, Austria; c Institute for Economic Research, Ljubljana, Slovenia; d Faculty of Economics, University of Ljubljana, Ljubljana, Slovenia

**Keywords:** Macroeconomics, fiscal policy, Slovenia, crisis, public debt

## Abstract

In this article, we use the macroeconometric model SLOPOL10 to calculate simulations of the development of the Slovenian economy until 2030. Starting from the present favourable prospects of the European economies, the forecast is very optimistic but it can nevertheless be improved by optimal fiscal policies as calculated using the OPTCON2 algorithm. If a negative shock to world trade of a size comparable to the Great Recession occurs, it will entail a decline in GDP and a slow recovery. In this case, optimal fiscal policies should not act in an expansionary way as the effectiveness of fiscal policy with respect to output and employment is rather limited in a small open economy like Slovenia. Instead, the goal of budget consolidation will call for a more restrictive fiscal policy, at least if the shock is temporary.

## 1. Introduction

The financial and economic crisis of 2007–2009, now commonly dubbed the Great Recession, hit Slovenia particularly hard, with real GDP dropping by almost 8% in 2009. Furthermore, Slovenia experienced a double-dip recession, since after a temporary recovery GDP contracted again in 2012 and 2013. As a result, the harmonised unemployment rate more than doubled from a low of 4.4% in 2008 (annual average) to 10% in 2013, before it started to decline again due to the emergence of an economic recovery.

Before the Great Recession, Slovenia had experienced high GDP growth and falling unemployment. However, this seemingly positive development hid underlying problems, among them formation of real estate, construction and stock market bubbles, managers’ buyouts during the second wave of privatisation of state-owned companies, growing systemic risk based on under-capitalisation of banks and excessive risk taking in the banking sector, and wage growth in excess of labour productivity growth due to the aggressive behaviour of trade unions and employers’ organisations (see e.g. Brezigar Masten et al., 2014; Verbič, Srakar, Majcen, & Čok, 2016). Slovenia entered the European Union in 2004 and the Euro Area in 2007, which is part of the explanation for the relative availability of loans as a stimulus which lead to the formation of the ‘bubbles’. Upon the outbreak of the Great Recession, these structural problems came to the surface. The banks were faced with liquidity problems, and confronted with the sudden loss of external financing, many companies turned out to be insolvent or close to insolvency. This led to widespread bankruptcies, an inability to service debt and mounting non-performing loans, ultimately resulting in sharply declining bank capital (IMF, 2017).

The necessary injections of public money aggravated the increase in government debt, which was already rising sharply due to the working of automatic stabilisers. Due to large budget deficits over the last few years as well as the increased level of debt, Slovenia is currently subject to the preventive arm of the Stability and Growth Pact (SGP) and should attempt to make sufficient progress towards its medium-term objective (MTO). In addition, its debt ratio has to be reduced. According to the 2017 update of the Slovenian Stability Programme, the MTO should lead to a balanced budget being achieved in 2020. However, as the European Commission notes in its assessment of the Stability Programme, this target is below the minimum MTO for Slovenia, namely 0.25% of GDP (European Commission, 2017). Hence, there is the need for a more ambitious consolidation of public finances.

**Figure 1.**
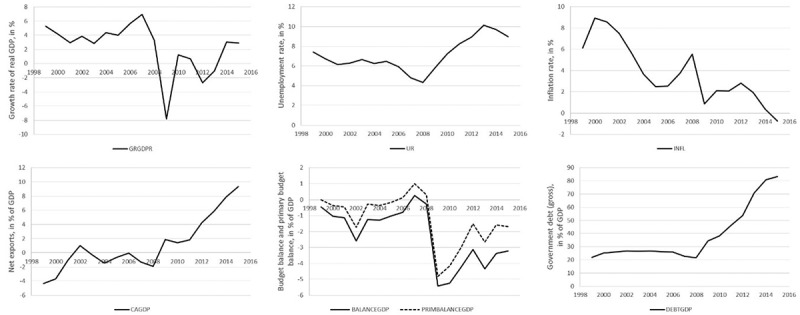
Dynamics of main macroeconomic aggregates in Slovenia, 1998–2016. Top left: growth rate of real GDP; top middle: unemployment rate; top right: inflation rate; bottom left: net exports; bottom middle: budget balance and primary budget balance; bottom right: government debt.

In Figure 1, a summary presentation of the dynamics of main Slovenian macroeconomic aggregates in the period 1998–2016 is presented. After a brief transformational recession in 1991–1992, high real GDP growth rates were achieved until 2008, and per capita income converged to the EU average faster than in any other transitional country in Central and Eastern Europe. According to Okun’s law, the high growth rates corresponded to a falling unemployment rate, which in 2008 reached its lowest point at 4.3%. Despite the decline in the unemployment rate, the rate of inflation initially dropped, although most recently the rise in inflation to 5.5% indicated the problem of possible overheating. The current account was broadly balanced, as was the primary balance of the general government budget, while the actual budget balance was in deficit, but always below the ‘Maastricht limit’ of 3% of GDP. As a result, government debt remained essentially stable in relation to GDP and far below the 60% of GDP allowed under the EU Stability and Growth Pact (Neck, 2017, pp. 96–97).

In this article, we analyse the effects of different fiscal policy scenarios in Slovenia over the next 15 years and evaluate them according to their effects on macroeconomic target variables. We also determine optimal fiscal policies for Slovenia and, using the OPTCON algorithm, calculate approximately optimal policies under different scenarios. These analyses highlight trade-offs between the design of countercyclical fiscal policies and the requirements of fiscal solvency and point to the current projections and future paths for solving the pressing public finance situation in Slovenia.

The significant increase in public debt as a legacy of the crisis – partly due to a weak macroeconomic policy response to the crisis – may also be of interest to other former Yugoslav countries that intend to become members of the European Union. One may conjecture that even a successful transformation to a market economy is not sufficient for compliance with EU standards in the case of a severe global economic disturbance. For instance, public finances may be insufficiently prepared to deal with a sharp drop in aggregate demand if political authorities are under pressure from trade unions to pay continuously excess wages to public employees. Therefore, we are interested in how fiscal policies should be designed in a country like Slovenia in order to reduce public debt without affecting its macroeconomic performance.

A large body of literature has been published in the last couple of years on the role of fiscal policies and the specific problems of countries within the Euro Area (see, for instance, Alesina, Azzalini, Favero, Giavazzi, & Miano, 2018; Coenen, Mohr, & Straub, 2008; Cogan, Cwik, Taylor, & Wieland, 2010; Martin, 2018; Romer & Romer, 2010; Taylor, 2009). It is well known that fiscal policy effects are smaller ceteris paribus in small open economies than in larger economies that are less open, but the empirical evidence is also mixed for open economies. Slovenia is an interesting case because it is a small open transition economy that was already in the Euro Area before the Great Recession.

To analyse the effects of different fiscal policy scenarios in Slovenia over the next 15 years and evaluate them, we use the SLOPOL model, an econometric model of the Slovenian economy constructed by the authors of this article to make forecasts and simulate the effects of the global and European crises under alternative assumptions. Next, we determine optimal fiscal policies for Slovenia, using the SLOPOL model and assuming an intertemporal objective function for Slovenian policy makers containing output, unemployment, inflation, the budget deficit, public debt and the current account as arguments. Using the OPTCON algorithm, approximately optimal policies are calculated under different scenarios. We analyse both demand side and supply side orientated policies.

The article is structured as follows. In the next section, we present the construction and basic parameters of the SLOPOL10 model. Long-run projections of the Slovenian economy are presented in Section 3. Optimal control approach and its results for optimal fiscal policies in Slovenia are presented in Sections 4 and 5. Simulations under an alternative crisis scenario are presented in Section 6. Section 7 concludes with a presentation of the main findings.

## 2. The SLOPOL10 model

For the simulations and optimisations, we used SLOPOL10, a macroeconometric model of the small open economy of Slovenia. The current version consists of 75 equations, of which 23 are behavioural and 52 are identities. In addition, the model contains 41 exogenous variables. The behavioural equations were estimated by ordinary least squares (OLS), except for the labour demand and supply equations, which were estimated as censored Tobit models. Almost all behavioural equations were specified in error correction form. The equations are based on quarterly data for the period 1995q1–2015q4. The current version of the model is built on earlier versions as described in Neck, Blueschke, and Weyerstrass (2011). For the present article, we updated the time series, re-estimated the equations and made some amendments to the model. In this section, the model equations are described very briefly. A detailed description, along with a list of the equations and variables, is provided in Weyerstrass et al. (2018).

In some equations the statistical tests point to serial correlation of the error terms. As in most if not all structural econometric models in use by central banks or research institutes around the world (for related considerations, see e.g. Grech & Rapa, 2016), statistical soundness was a key, but not the exclusive concern when selecting behavioural equations among alternative specifications. In some cases, therefore, the selection of the final specification of an equation involved some trade-off between statistical soundness and desirable simulation properties or theoretical consistency. Moreover, our dataset has at most 20 years of data, and the time series include the period during which the Slovenian economy underwent the transition towards a market economy, the EU accession, the Euro Area accession, and finally the Great Recession and the following public debt crisis. Hence, theoretical soundness and model simulation properties were given priority when selecting the equations.

The model is based on the aggregate supply/aggregate demand (AS/AD) framework. Hence, it contains equations for the supply side and the demand side. Rigidities of wages and prices are taken into account. The model combines Keynesian and neoclassical elements, the former determining the short and medium run solutions in the sense that the model is demand-driven and persistent disequilibria in the goods and labour markets are possible.

On the supply side, potential output is determined via a Cobb–Douglas production function with constant returns to scale. Potential GDP depends on trend employment, the capital stock and autonomous technical progress. Ex post, total factor productivity (TFP) is calculated as the Solow residual. In a second step, the trend of technical progress is determined by applying the HP filter. For simulations and forecasts, the trend of the TFP is explained in a behavioural equation with the proportion of people with tertiary education in the labour force, the real investment ratio, i.e. gross fixed capital formation over GDP, and lagged real government spending on research and development (R&D) as explanatory variables. Although one might argue that the equation explaining TFP, which is non-stationary, should be specified in growth rates, we used a specification in levels. In the literature, both approaches can be found. Harris and Moffat (2015) as well as Dettori, Marrocu, and Paci (2011) also analyse the determinants of TFP by a regression in levels. Some of the explanatory variables, in particular the share of people with tertiary education, show little variation in the data, rendering a specification in growth rates difficult. In addition, further statistical analyses identify the variables used in the TFP equation as cointegrated; hence, the specification in levels seems appropriate.

On the demand side, private consumption, gross fixed capital formation, exports and imports are determined in behavioural equations. On the labour market, labour demand and supply are divided into the main age group (15–64 years) and older people (65 years and above). Labour demand (actual employment) is modelled via the employment rates of the two age groups, i.e. employment as a proportion of the relevant age groups. Both employment rates are influenced positively by real GDP and negatively by the real net wage and additionally by the wedge between the gross and the net wage. Labour supply is modelled via the proportion of the labour force of the two age groups in the total population. It depends positively on the real net wage and negatively on the wedge between the gross and the net wage.

In the wage–price system, gross wages, the CPI and various deflators are determined. On the money market, the short-term interest rate, the long-term interest rate and the real effective exchange rate are determined. The government sector block contains equations for social security revenues, profit taxes, value added tax (VAT) revenues, interest payments on public debt, and other revenues and expenditures. The following fiscal policy instruments are considered: government consumption, transfers to private households, public investment on machinery and construction, government expenditure on R&D, the VAT rate, the personal income tax rate and the social security contribution rate. In addition, we treat the proportion of the labour force with tertiary education as a policy instrument, although it is not under the direct control of the government.

Just like any structural macroeconometric model, the SLOPOL model is subject to the Lucas (1976) critique according to which the relations between macroeconomic aggregates in an econometric model should differ according to the macroeconomic policy regime in place. An approach taking the Lucas critique into account in structural models like SLOPOL emerged in the so-called London School of Economics tradition initiated by Sargan (1964). According to this approach, economic theory guides the determination of the underlying long-run specification, while the dynamic adjustment process is derived from an analysis of the time series properties of the data series. Error correction models involving cointegrated variables combine the long-run equilibrium and the short-run adjustment mechanism.

## 3. A long-run projection of the Slovenian economy

We analyse the macroeconomic and fiscal performance of the Slovenian economy over the period 2017–2030. To this end, we first run a projection of the SLOPOL model, which requires assumptions regarding the exogenous variables. Since the model is based on data until 2015, our projection has to start in 2016, but when interpreting the results we focus on the period starting in 2017. The simulation requires assumptions on the exogenous variables. The exogenous variables comprise those (totally or largely) outside the influence of Slovenian policy makers (e.g. world trade, oil price, exchange rate, Euro Area interest rates, population development) and the fiscal policy instruments. These assumptions and the results of the baseline simulation are described in this section. Afterwards, we determine optimal policies, i.e. we let an optimisation algorithm determine the paths of the policy instruments required to come as close as possible to pre-specified ‘ideal values’ of target variables.

Table 1 shows the assumed paths of the fiscal policy instruments: public consumption according to fiscal statistics, nominal (*GNFIN*), transfers to individuals and households (*TRANSFERSN*), remaining government expenditures (*REVREST*), public investment, nominal (GINVN), public expenditures on research and development (*GERD*), average personal income tax rate (*INCTAXRATE*), average social security contribution rate (*SOCEMPRATE*), value added tax rate (*VATAXRATE*) and active working population with tertiary education, % of total (*LFTERSHARE*). As we had to start the simulation in 2016, assumptions also had to be made for 2016 and 2017 for which now – at least provisional – data are available. Thus, the table also shows the actual realisations of the instruments in 2016 and 2017. For government expenditures on research and development, data for 2017 are not yet available.

**Table 1.** Assumptions for fiscal policy instruments.

	Assumptions		Actual development	
	2016	2017–2030	2016	2017
*Growth rates*				
*GNFIN*	1.1%	3.5%	4.0%	6.6%
*TRANSFERSN*	3.5%	3.5%	2.0%	2.6%
*REVREST*	3.5%	3.5%	−16.2%	−0.5%
*GINVN*	2.1%	3.5%	−29.2%	3.5%
*GERD*	3.5%	3.5%	1.8%	n.a.
*Absolute values*				
*INCTAXRATE*	12.7%	12.7%	11.9%	11.7%
*SOCEMPRATE*	18.2%	18.2%	17.4%	17.2%
*VATAXRATE*	22.0%	22.0%	22.0%	22.0%
*LFTERSHARE*	32.8%	incr. to 39.5%	33.9%	35.1%

As mentioned, in addition to the policy instruments, assumptions had to be made for the truly exogenous variables (those not controlled by the government of Slovenia). They are summarised in Table 2. Here, realisations for 2016 and 2017 are shown in parentheses. In addition to the variables in the table, population projections were needed. According to current projections,^1^ Slovenia’s working-age population will decline by around 0.75% per year until 2022 and by around 0.5% per year after that. Conversely, as is the case all over Europe, the population aged 65 and over will continue to rise due to increased life expectancy. According to population projections, this growth will decrease more or less steadily from about 3% in 2017 to 1.5% in 2030.

**Table 2.** Assumptions for the exogenous variables.

	2016	2017	2018–2030
World trade growth (%)	1.1 (1.5)	1.8 (4.5)	3.0
Oil price (USD/barrel)	42.56 (45.06)	53.63 (54.80)	incr. to 75
3 months Euribor (%)	−0.27 (−0.27)	−0.25 (−0.33)	incr. to 0.75
10 year Euro Area gov. bond yield (%)	0.87 (0.87)	1.01 (1.09)	incr. to 2.00
USD/EUR	1.11 (1.11)	1.09 (1.13)	1.10

These settings of the exogenous variables lead to the baseline simulation results as summarised in Table 3. As can be seen, our model underestimated the rather fast recovery in 2017 from the previous slow and stagnating development of the Slovenian economy and the return to lower public debt.

**Table 3.** Results of the baseline simulation (for 2016 and 2017, actual realisations are shown in parentheses).

	Real GDP growth	Employment growth	Unemployment rate	Inflation rate	Budget balance/GDP	Public debt/GDP
2016	2.1 (*3.1*)	0.9 (−*0.3*)	8.3 (*8.0*)	−0.5 (−*0.2*)	−2.3 (−*1.6*)	82.6 (*78.5*)
2017	2.9 (*5.0*)	0.9 (*4.8*)	7.2 (*6.6*)	−0.2 (*1.6*)	−2.1 (−*0.7*)	82.2 (*73.5*)
2018	3.3	0.5	6.3	−0.1	−1.9	81.2
2019	3.1	0.3	5.5	0.3	−1.6	79.8
2020	3.0	0.2	4.9	0.6	−1.4	78.1
2021	2.8	0.1	4.3	0.9	−1.3	76.3
2022	2.7	0.2	3.6	1.0	−1.3	74.7
2023	2.4	0.0	3.3	1.1	−1.2	73.3
2024	2.4	0.0	3.0	1.2	−1.1	71.8
2025	2.3	−0.1	2.8	1.2	−1.0	70.4
2026	2.2	−0.1	2.5	1.1	−1.1	69.1
2027	2.2	−0.1	2.3	1.1	−1.2	68.0
2028	2.2	−0.1	2.1	1.0	−1.3	67.1
2029	2.2	−0.1	1.8	1.0	−1.4	66.4
2030	2.2	−0.1	1.5	0.9	−1.6	65.8

## 4. The optimal control approach

In addition to the long-run projections as described in the previous section, we run several optimal control exercises to obtain optimal fiscal policy trajectories. Solving an optimum control problem means finding certain paths of control variables that minimise an objective function involving deviations of the values of the politically relevant variables from some pre-specified target paths. As usual in economic policy applications, we assume a quadratic objective function. The problem is described as follows:

(1) $$\min J=\;\sum_{t=1}^{T}L_{t}(x_{t},u_{t}),$$

$$L_{t}\left(x_{t,}u_{t}\right)=\frac{1}{2}{\left(\begin{array}{c} x_{t}-{\tilde{x}}_{t}\\ u_{t}-{\tilde{u}}_{t} \end{array}\right)}^{{\;}^{\prime }}W_{t}\left(\begin{array}{c} x_{t}-{\tilde{x}}_{t}\\ u_{t}-{\tilde{u}}_{t} \end{array}\right).$$

Here $x_{t}$ is an *n*-dimensional vector of state variables that describes the state of the economic system at time *t*; $u_{t}$ is an *m*-dimensional vector of control (policy instrument) variables; ${\tilde{x}}_{t}\in R^{n}$ and ${\tilde{u}}_{t}\in R^{m}$ are given ‘ideal’ (desired) levels of the state and control variables respectively. *T* denotes the terminal period of the finite planning horizon; $W_{t}$ is a matrix specifying the relative weights of the state and control variables in the objective function.

The optimisation is restricted by the dynamics of the system given in the form of a system of nonlinear difference equations:

(2) $$x_{t}=f(x_{t-1},x_{t},u_{t},\theta ,z_{t})+\varepsilon_{t},\;t=1,\ldots ,T,$$

where θ is a *p*-dimensional vector of estimated parameters and $z_{t}$ denotes a vector of exogenous non-controlled variables. In this study, the dynamic system *f* is given by the SLOPOL10 model.

The dynamic system (2) and the objective function (1) define a multivariable nonlinear-quadratic optimum control problem which has to be solved.

An exact solution to such a problem is not possible, so we have to resort to numerical approximations. To this end, the OPTCON2 algorithm is used (for more details see Blueschke-Nikolaeva, Blueschke, and Neck (2012)). This algorithm determines approximate solutions to optimum control problems with a quadratic objective function and a nonlinear multivariate dynamic system under additive and parameter uncertainties. Although this algorithm allows for a rather elaborate menu of stochastic extensions, here we confine ourselves to deterministic optimal control, assuming the model parameters and the model equations to be exactly true.

## 5. Optimal fiscal policies for Slovenia

The policy maker in this optimal control experiment is the government of Slovenia, which calculates the optimal trajectories of policy instruments until 2030. It has nine control variables at its disposal: government consumption, transfers, government investment, public expenditures for research and development, the average personal income tax rate, the proportion of the active working population with tertiary education, the average social security contribution rate, remaining government revenues and the value added tax rate. We selected 11 state variables for which certain ‘ideal’ paths are defined and which enter the objective function (1), namely the growth rate of GDP (*GRGDPR*), the level of real GDP (*GDPR*), the unemployment rate (*UR*), the inflation rate (*INFL*), the budget balance ratio to GDP (*BALANCEGDP*), the debt level ratio to GDP (*DEBTGDP*), the current account balance ratio to GDP (*CAGDP*), real private consumption (*CR*), real private investment (*PRINVR*), the growth rate of potential GDP (*GRYPOT*) and the level of potential GDP (*YPOT*). The target paths of the main objectives are shown in Table 4.

**Table 4.** Targets for the optimisations.

	Budget balance/GDP	Net exports/GDP	Public debt/GDP	Output gap	Real GDP growth	Potential GDP growth	Inflation rate	Unemployment rate
2017	−2.1%	9.4%	82.0%	−0.8%	3.0%	3.6%	−0.2%	7.1%
2018	0.0%	9.5%	78.0%	0.0%	3.0%	3.0%	1.0%	6.1%
2019	0.0%	9.4%	74.0%	0.0%	3.0%	3.0%	1.0%	5.1%
2020	0.0%	9.3%	70.0%	0.0%	3.0%	3.0%	1.0%	4.6%
2021	0.0%	9.2%	66.0%	0.0%	3.0%	3.0%	1.0%	4.1%
2022	0.0%	9.1%	62.0%	0.0%	2.6%	2.6%	1.0%	3.6%
2023	0.0%	9.0%	61.0%	0.0%	2.5%	2.5%	1.0%	3.4%
2024	0.0%	8.9%	60.0%	0.0%	2.5%	2.5%	1.0%	3.1%
2025	0.0%	8.8%	59.0%	0.0%	2.5%	2.5%	1.0%	2.9%
2026	0.0%	8.7%	58.0%	0.0%	2.5%	2.5%	1.0%	2.7%
2027	0.0%	8.6%	57.0%	0.0%	2.5%	2.5%	1.0%	2.5%
2028	0.0%	8.5%	56.0%	0.0%	2.5%	2.5%	1.0%	2.3%
2029	0.0%	8.4%	55.0%	0.0%	2.5%	2.5%	1.0%	2.1%
2030	0.0%	8.3%	54.0%	0.0%	2.5%	2.5%	1.0%	1.9%

The choice of targets is meant to represent the most important goals of macroeconomic policy making. The ‘ideal’ paths imply smooth growth in the income variables and low values for the rates of unemployment and inflation. In addition to the targets depicted in the table, for the levels of real GDP, potential output, private consumption and investment, target paths in accordance with the ‘ideal’ real growth rates had to be specified. Furthermore, ‘ideal’ paths had to be given to the instruments in order to prevent erratic fluctuations in these variables.

Regarding the choice of the weights of the objective variables (the matrix *W* in (1)), we take the simplest possibility of giving all variables the same weight of 1. Of course, these raw weights are normalised according to the time-series characteristics of the variables.

Using the specified targets and weights, we are able to carry out the optimal control exercise and to calculate optimal fiscal policies according to the assumptions made. The optimal paths of the control variables are given in Figures 2–10 and denoted by ‘opt_sc0’. The optimal paths of the state variables are given in Figures 11–16. In addition, the figures include the non-controlled (projected) simulation paths as described in Section 3 (denoted by ‘baseline’).

**Figure 2.**
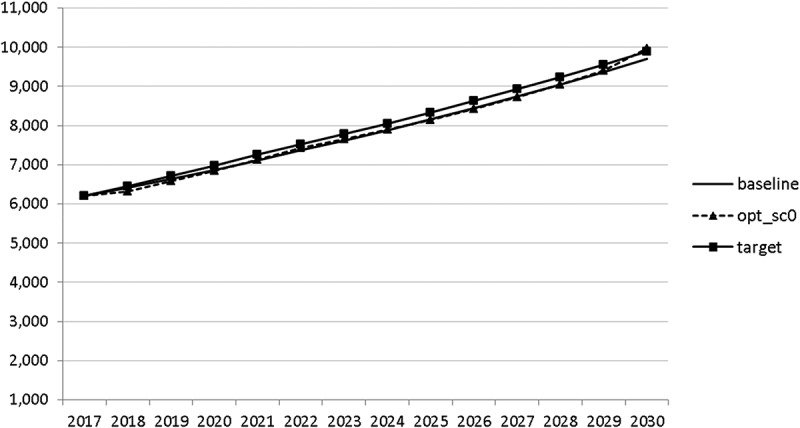
Government consumption (*GNFIN*), mio euro.

**Figure 3.**
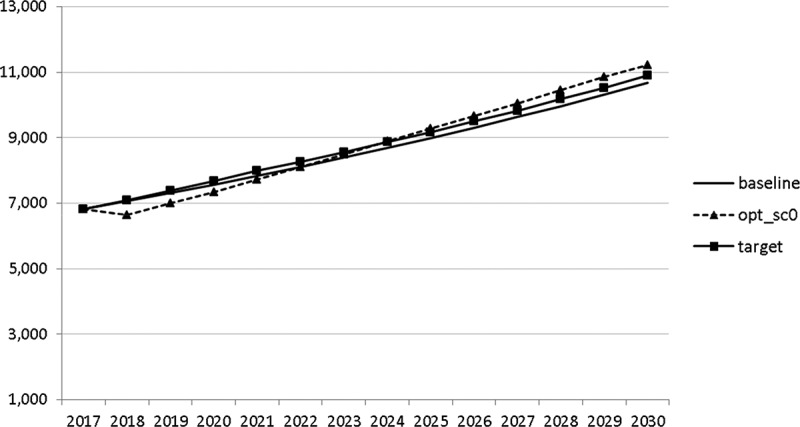
Transfers (*TRANSFERS*), mio euro.

**Figure 4.**
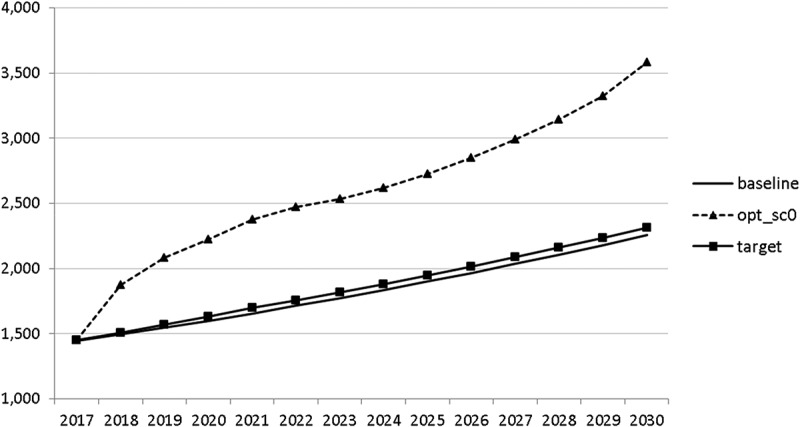
Government investment (*GINVN*), mio euro.

**Figure 5.**
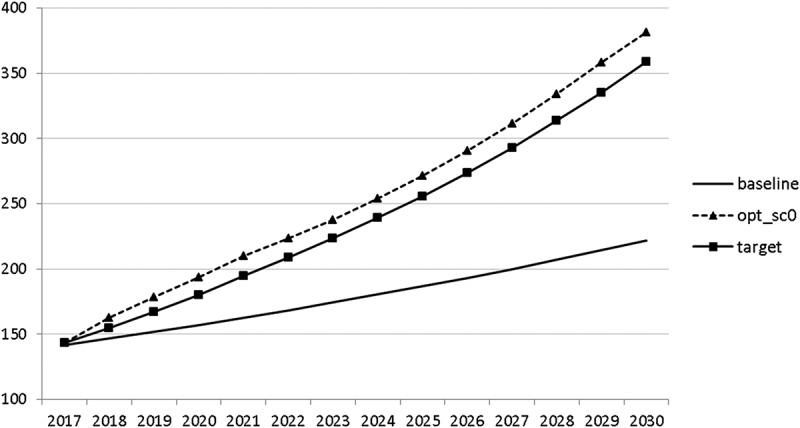
Public expenditures for R&D (*GERD*), mio euro.

**Figure 6.**
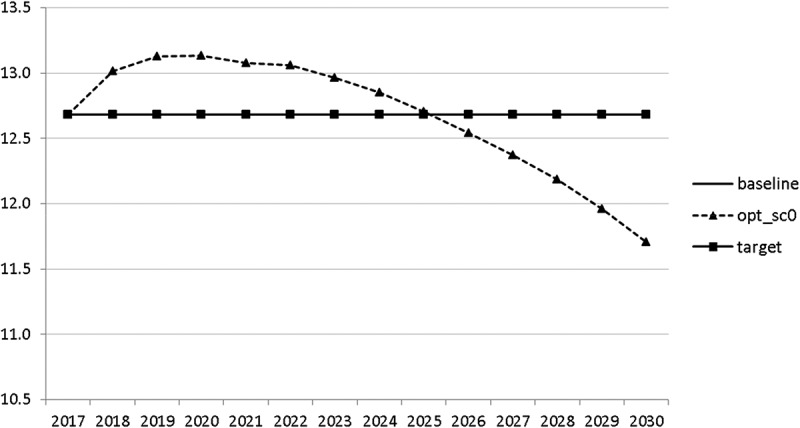
Average personal income tax rate (*INCTAXRATE*), %.

**Figure 7.**
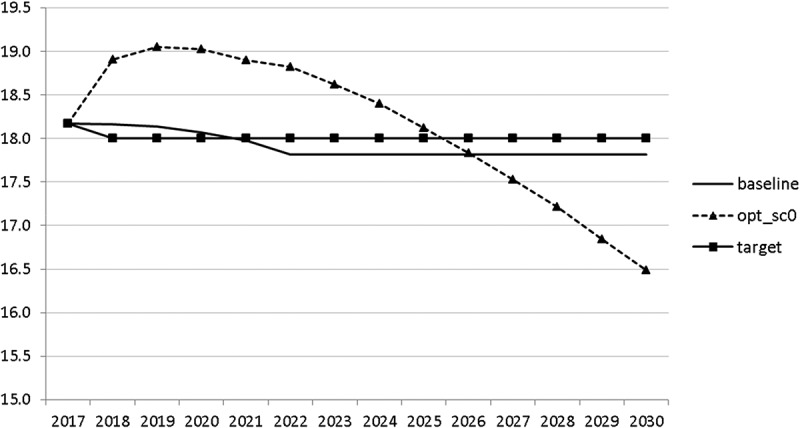
Average social security contribution rate (*SOCEMPRATE*), %.

**Figure 8.**
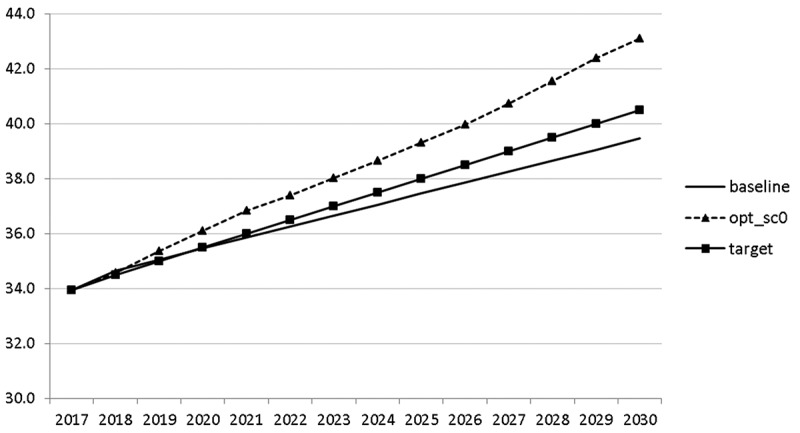
Active working population with tertiary education (*LFTERSHARE*), %.

**Figure 9.**
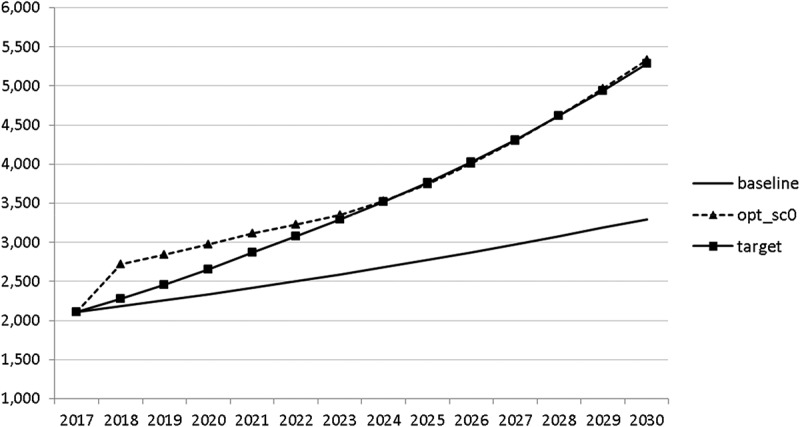
Remaining government revenues (*REVREST*), mio euro.

**Figure 10.**
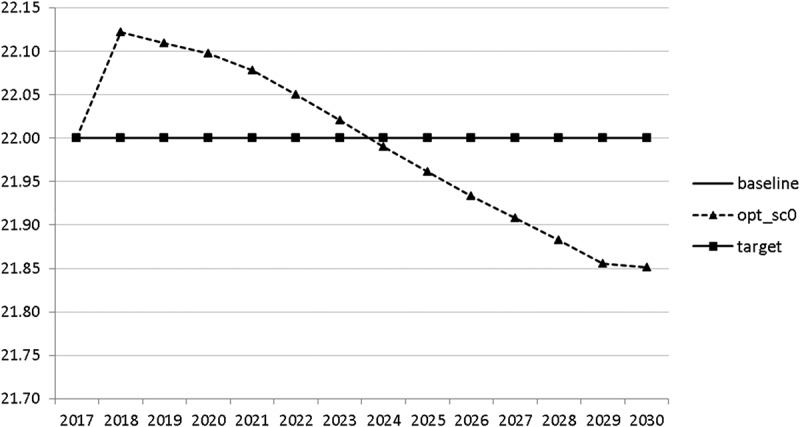
Value added tax rate (*VATAXRATE*), %.

**Figure 11.**
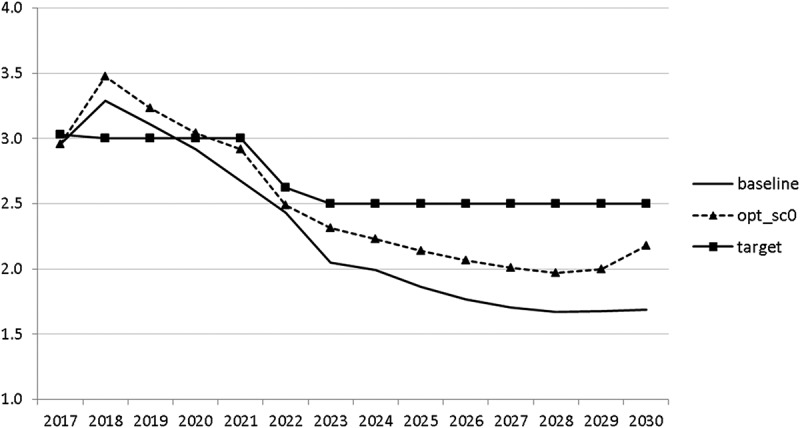
Growth rate of GDP (*GRGDPR*), %.

**Figure 12.**
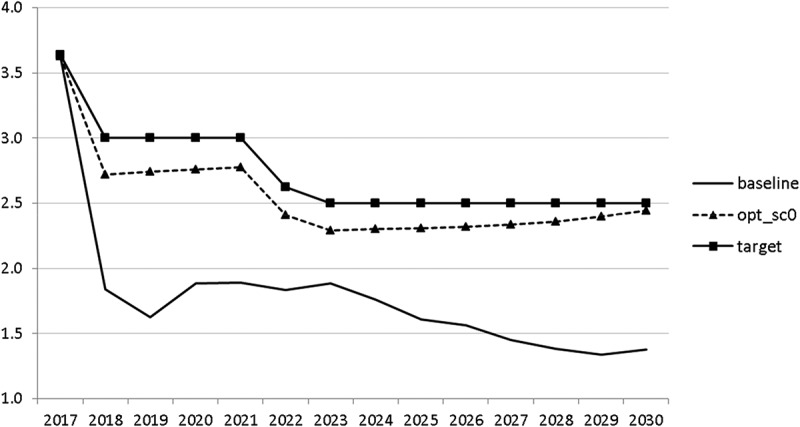
Growth rate of potential GDP (*GRYPOT*), %.

**Figure 13.**
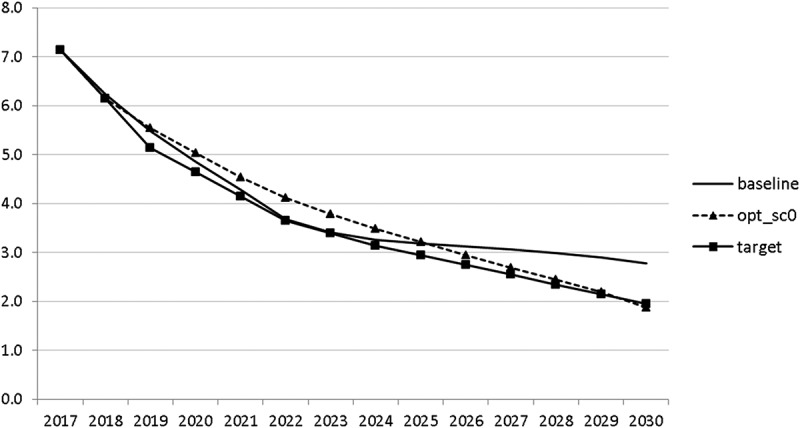
Unemployment rate (*UR*), %.

**Figure 14.**
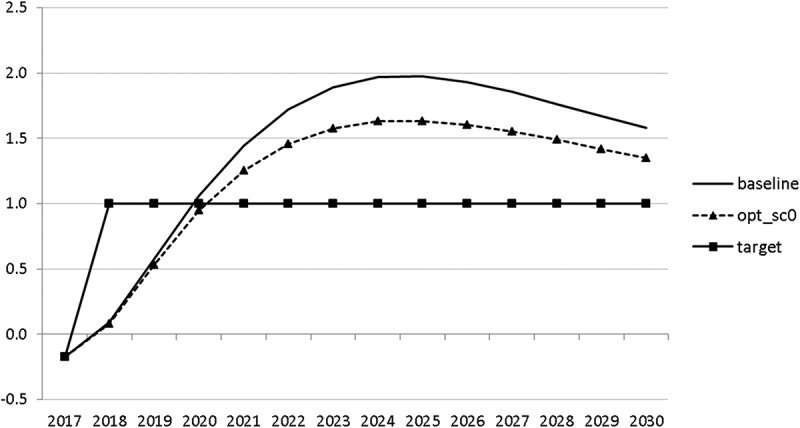
Inflation rate (*INFL*), %.

**Figure 15.**
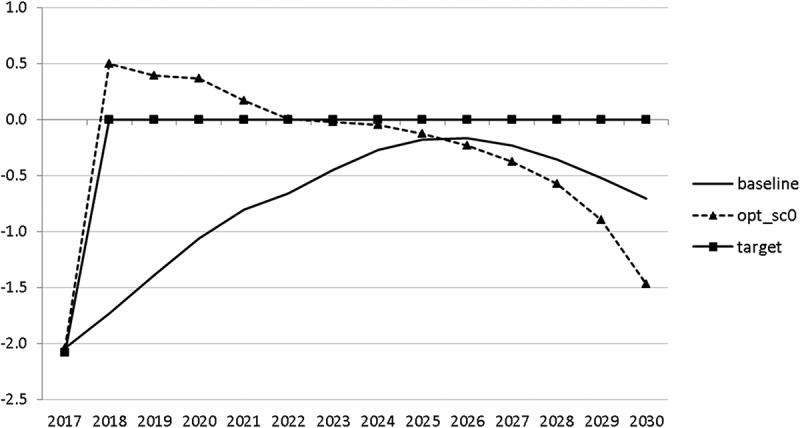
Budget balance ratio to GDP (*BALANCEGDP*), % of GDP.

**Figure 16.**
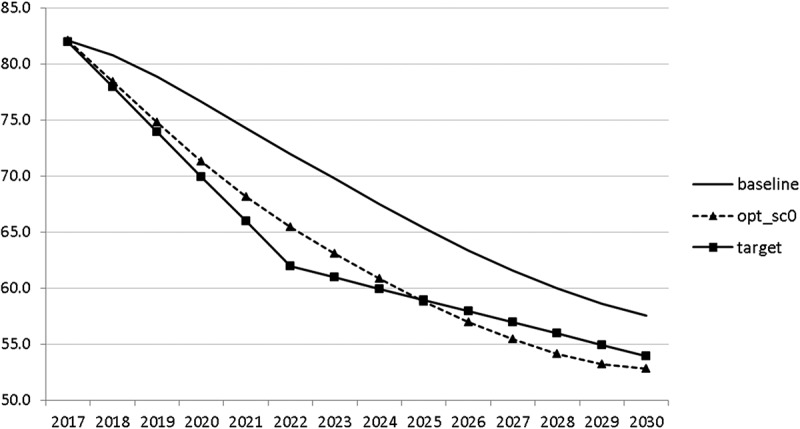
Public debt (*DEBTGDP*), % of GDP.

The calculated optimal policy mix is more or less obvious here, with a more restrictive fiscal policy in the first 5–6 years and a more expansionary policy afterwards. This is due to a relatively high level of debt and the necessity for budget consolidation. Two aspects attract attention: first, the optimal choice of budgetary policy entails a significant shift in priorities from transfers and general government consumption towards public investment, R&D related and education related expenditures. *GERD*, for example, grows from 150 mio euro to nearly 400 mio euro and *GINVN* should be increased by a factor of two. Compared to other fiscal instruments, these instruments also affect the supply side (*YPOT*), are more effective with respect to aggregate output and should be used more actively by the government. Second, quite often the optimal paths are very close to the non-controlled simulation, which shows that the Slovenian Stability Programme, which calls for budget consolidation, seems to be a reasonable choice for fiscal policy. This is supported by the resulting paths of the state variables.

The resulting paths of the state variables show a favourable forecast for the Slovenian economy. Despite a slightly restrictive fiscal policy in the next 5–6 years, GDP grows by around 3% per year. The unemployment rate decreases steadily, which is due to both the positive economic performance and the declining working-age population; it arrives at the desired 2% level in 2030. The inflation rate starts from a very low level and increases steadily until 2025 but stays below the ECB threshold of 2%. The budget is nearly balanced, with small surpluses in 2018–2021 and small deficits in 2025–2030. Although the highest budget deficit is achieved at the end of the planning horizon, it is only 1.5% of GDP. Relatively high growth rates of GDP and nearly balanced budgets allow the policy makers to deal with the problem of the high initial public debt relatively quickly. Already in 2025, a debt level below 60% of GDP is achieved. In the non-controlled baseline scenario, this happens in 2028.

Altogether, the optimal solution and the non-controlled projections show a very fortunate picture of the development of the Slovenian economy. As a small open economy, Slovenia is heavily dependent on external factors. The economic outlook for Slovenia, which was very pessimistic a few years ago, has changed a lot as the European economy shows strong upward dynamics. The Economic Sentiment Indicator (ESI), which describes the economic environment in Europe, is now at 111.9 points, the highest value in the last 10 years. However, as was shown by the optimisation results, it is important to shift some resources from sectors that are less to those that are more dynamic by increasing, for example, R&D-related and education-related expenditures.

## 6. What to do if a new crisis appears?

**Figure 17.**
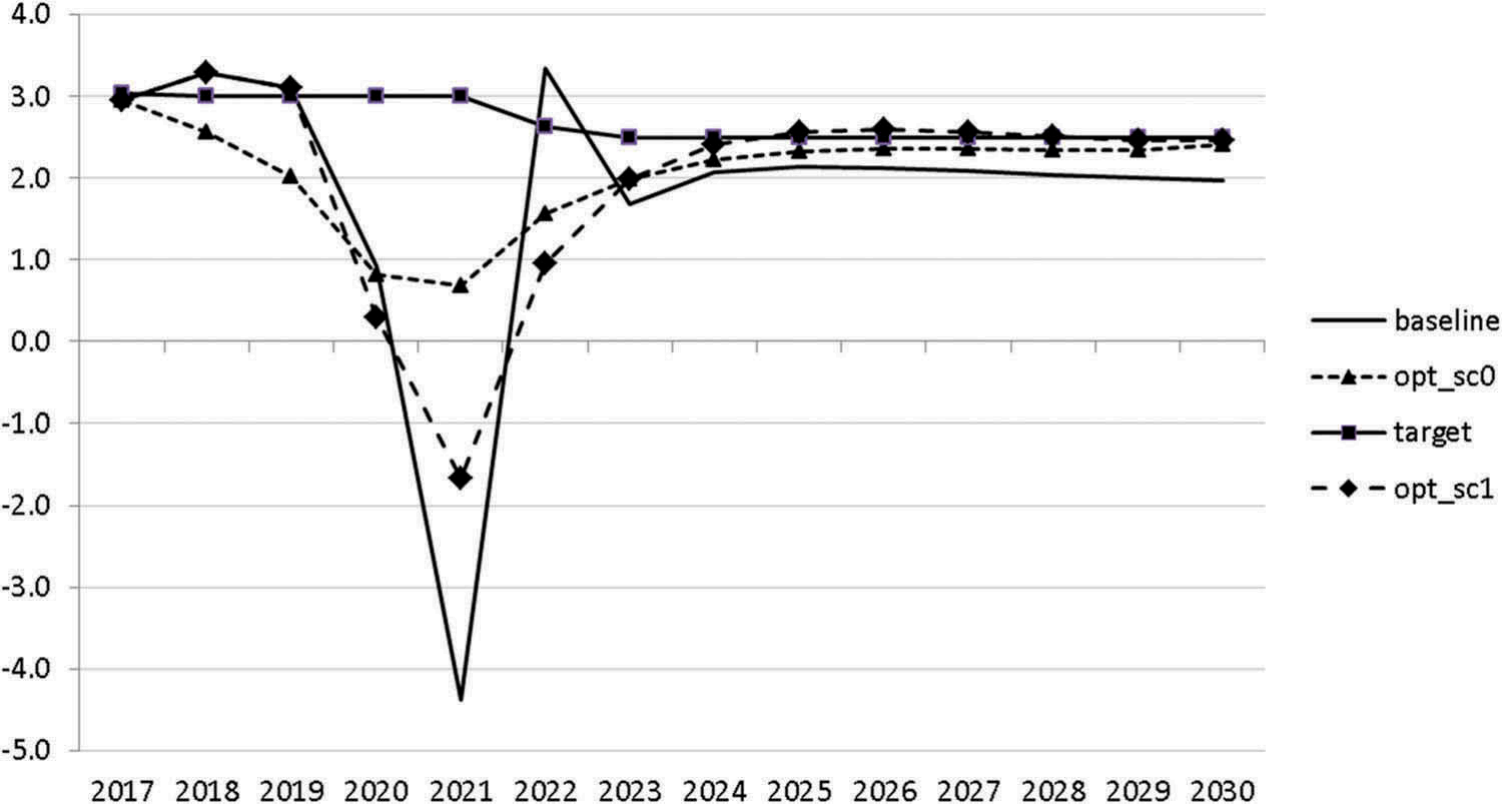
Growth rate of GDP (*GRGDPR*), %.

In Section 5, a relatively optimistic picture was presented for the Slovenian economy. This goes in line with the upward development of the European economy. However, it is important to be prepared for bad times. In this section, we analyse a situation in which a similar crisis to that of 2008–2010 occurs. We model this crisis by introducing a drop in world trade growth. We assume that the crisis breaks out in 2020. Starting in 2020q3 and continuing until the end of 2021, we calculate world trade by using the same growth rates as between 2008q3 and 2009q4, namely 2.5, −6.2, −17.7, −17.7, −12.9, −1.1. Using the adjusted world trade, we recalculate the uncontrolled projection (denoted again by ‘baseline’) and calculate two optimal scenarios. In scenario ‘opt_sc0’ we assume that the government already predicts in 2018 that a crisis will occur and calculate the optimal fiscal response for that case. In scenario ‘opt_sc1’ the outbreak of the crisis in 2020 is not predicted by the government. In Figure 17 the resulting paths are given for the growth rate of GDP in these scenarios.

In the uncontrolled projection, the outbreak of a new crisis in 2020 leads to a drop in GDP by more than 4%. If the government reacts to it properly, it is able to mitigate this negative effect to a large extent. Of course, this mitigation is much smoother if the government expects such a crisis (in 2021 *GRGDPR* is 0.7 in the opt_sc0 scenario as compared to −1.7 in the opt_sc1 scenario). In the first instance, anticipating the crisis does not seem to be a realistic assumption, but having experienced the Great Recession a few years ago, the government might learn from it and could immediately adjust its policy by reacting to the first signs of a bursting bubble.

In Figures 18–26, we present these optimal trajectories of the fiscal instruments.

**Figure 18.**
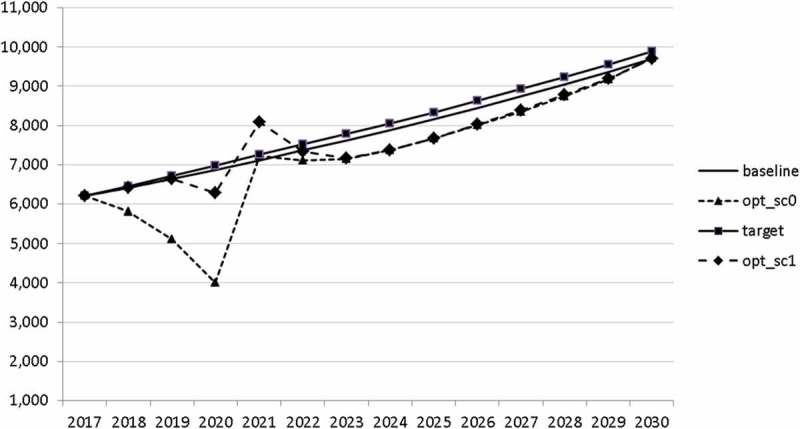
Government consumption (*GNFIN*), mio euro.

**Figure 19.**
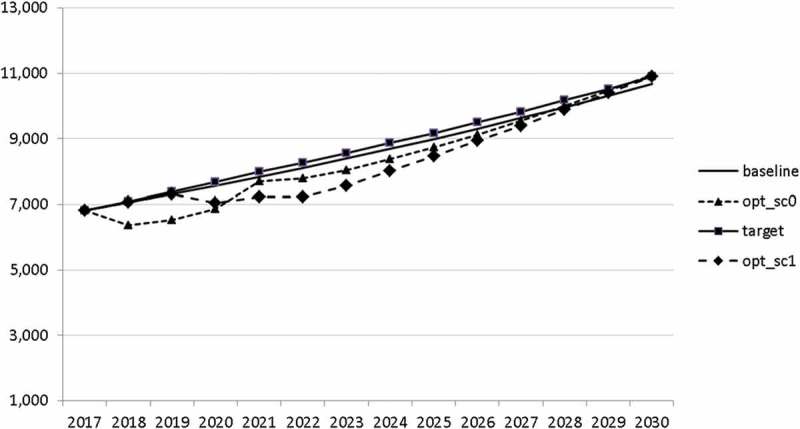
Transfers (*TRANSFERS*), mio euro.

**Figure 20.**
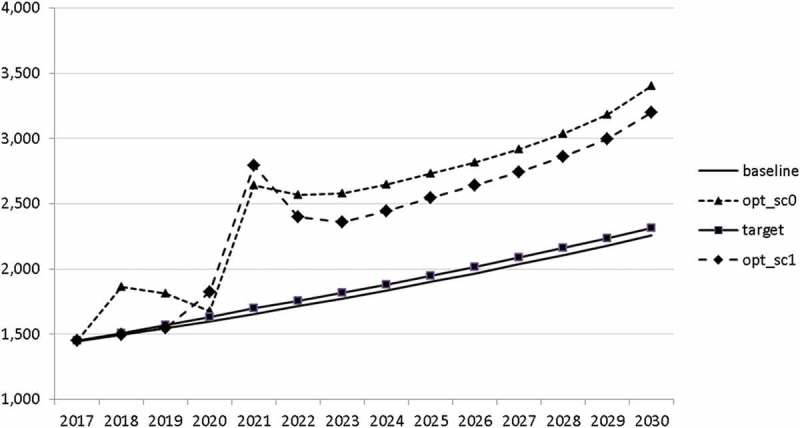
Government investment (*GINVN*), mio euro.

**Figure 21.**
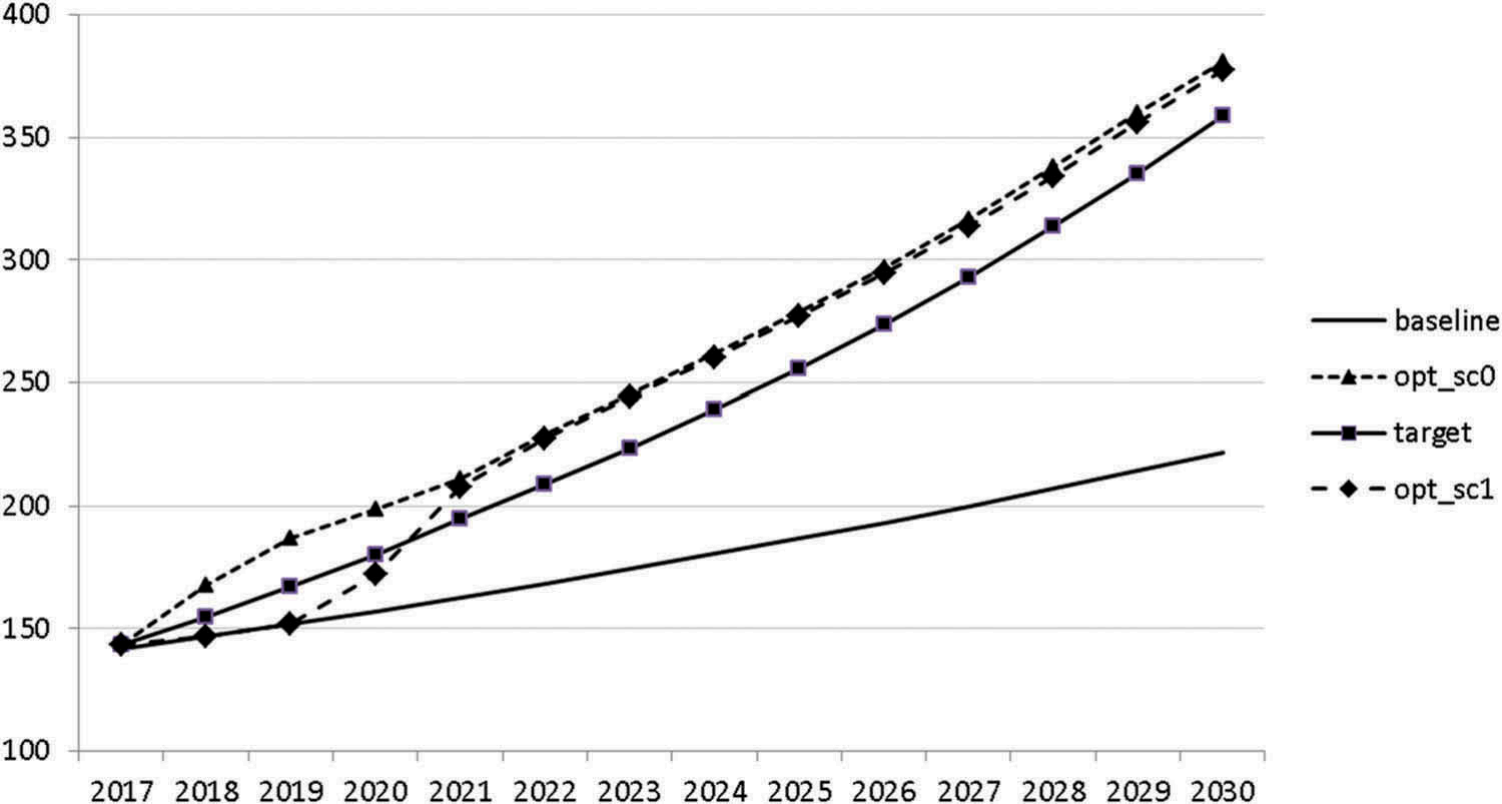
Public expenditures for R&D (*GERD*), mio euro.

**Figure 22.**
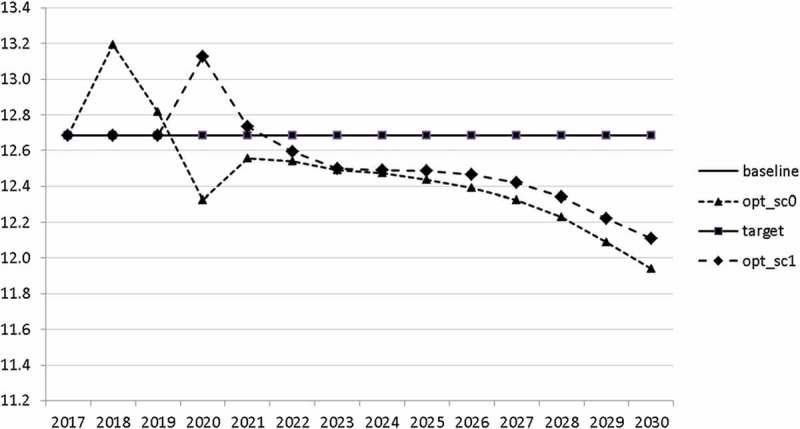
Average personal income tax rate (*INCTAXRATE*), %.

**Figure 23.**
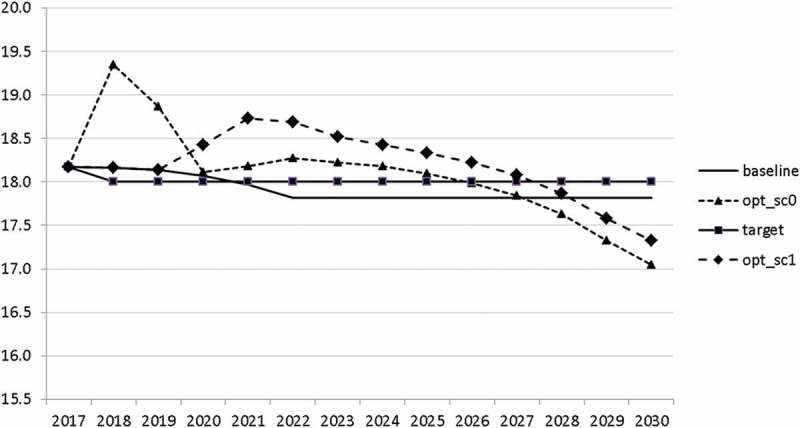
Average social security contribution rate (*SOCEMPRATE*), %.

**Figure 24.**
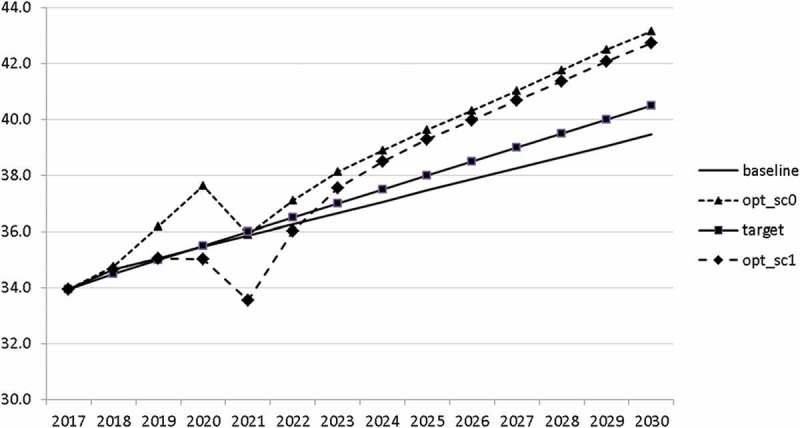
Active working population with tertiary education (*LFTERSHARE*), %.

**Figure 25.**
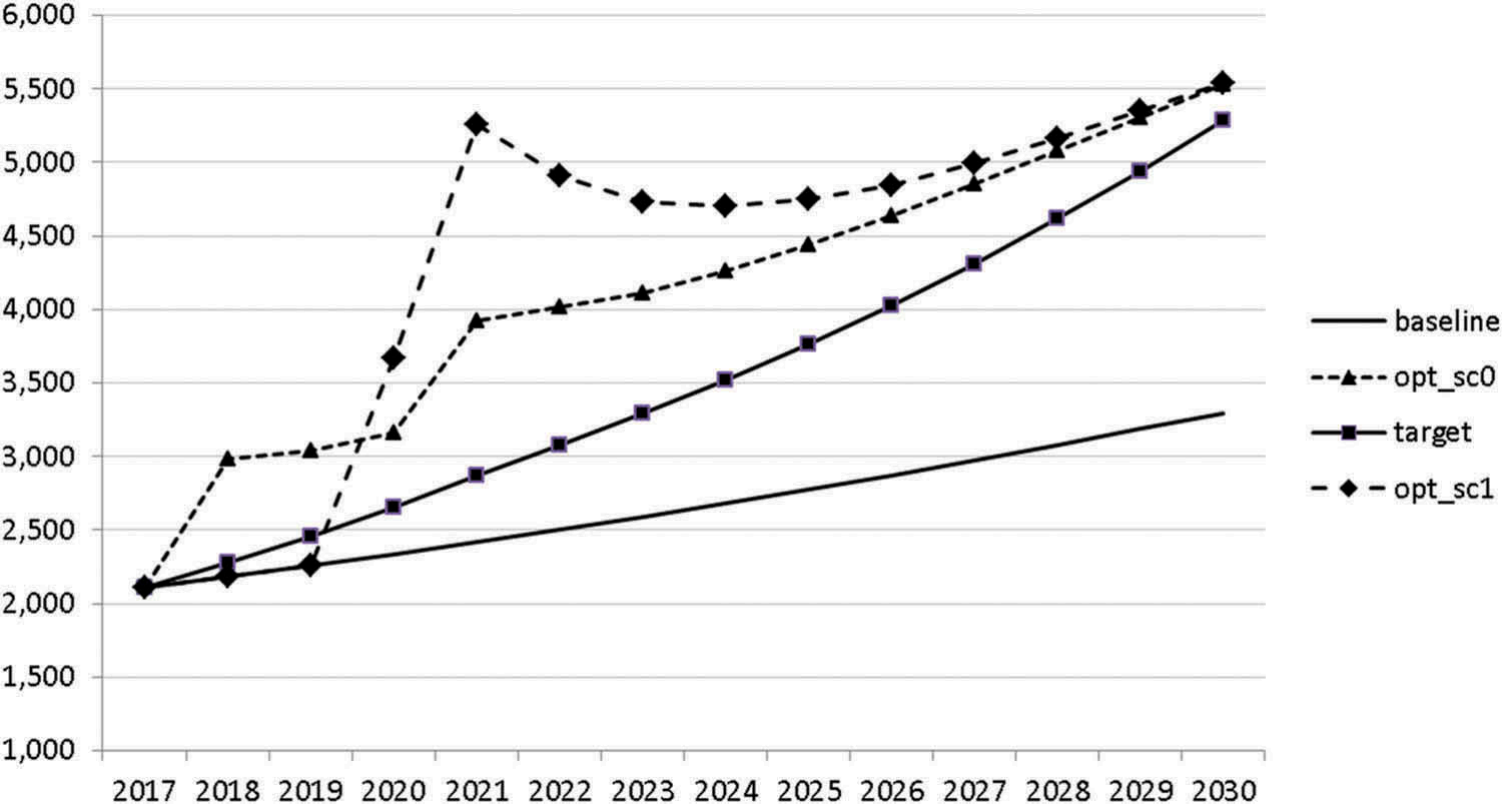
Remaining government revenues (*REVREST*), mio euro.

**Figure 26.**
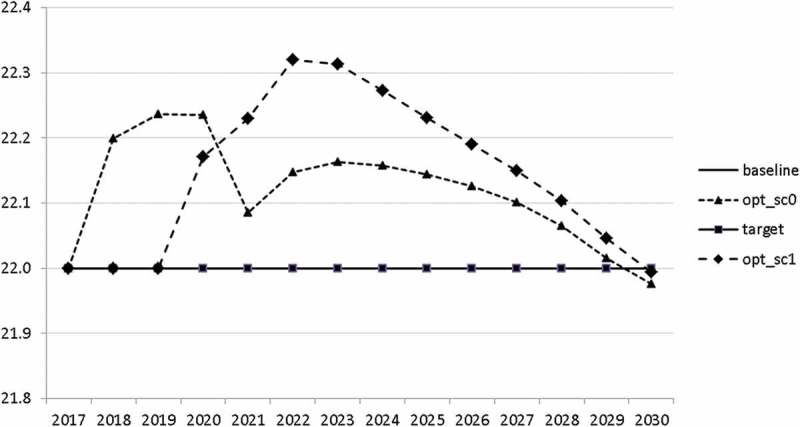
Value added tax rate (*VATAXRATE*), %.

Interestingly, the main difference between the scenarios with the negative shock and the scenario without the shock is a more restrictive stance on fiscal policy as shown by all the controls with the exception of public investment and expenditures for R&D and for human capital. The latter shows again the importance for output of supply side government expenditures. If the shock is expected, the government is asked to run a more restrictive fiscal policy right from the start. This is done to consolidate the budget as soon as possible and to be able to apply a more countercyclical fiscal policy during the crisis. In contrast, if the shock is unexpected, the government is required to be more restrictive even during the crisis, resulting in a larger drop in GDP.

**Figure 27.**
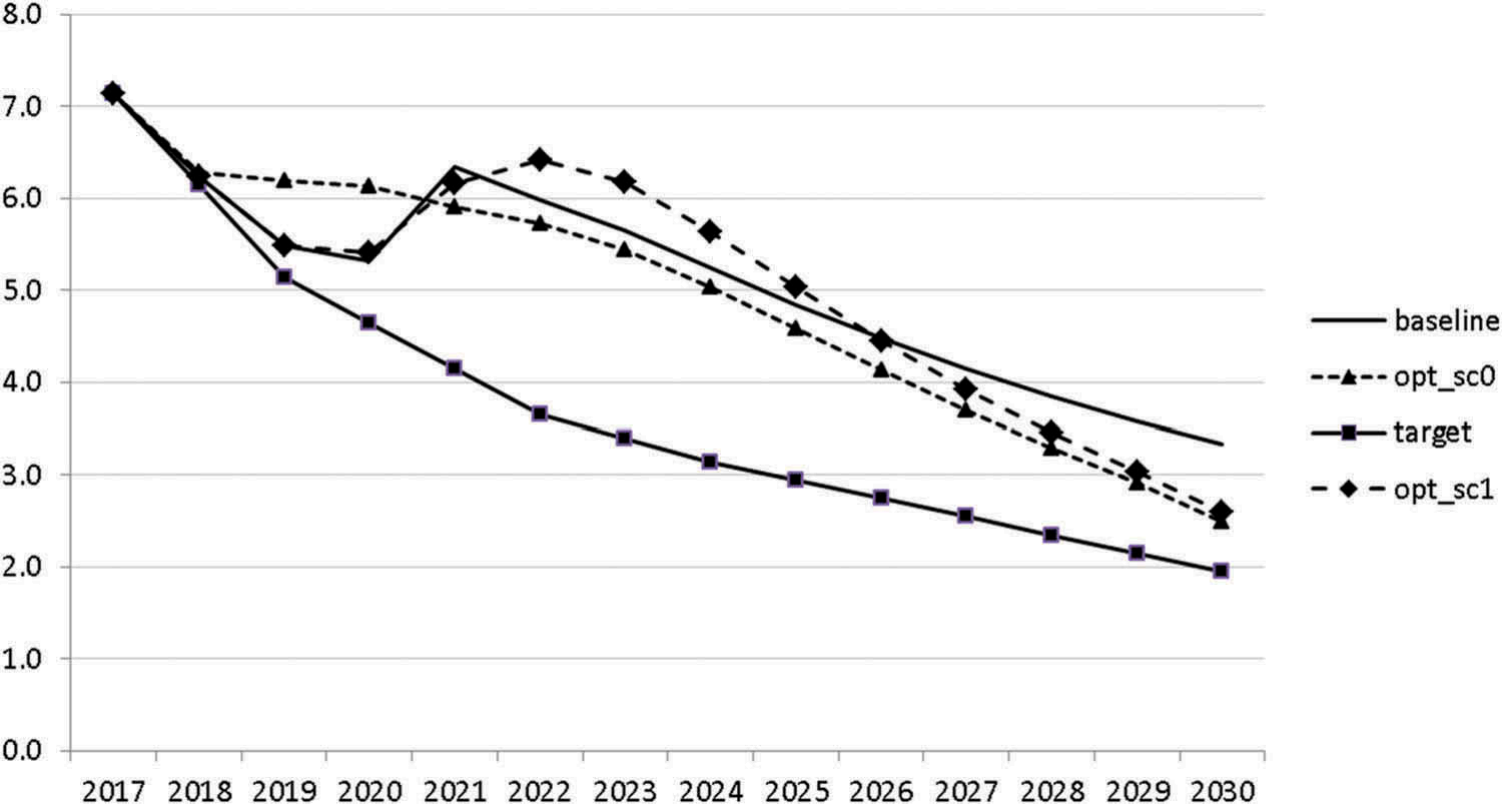
Unemployment rate (*UR*), %.

**Figure 28.**
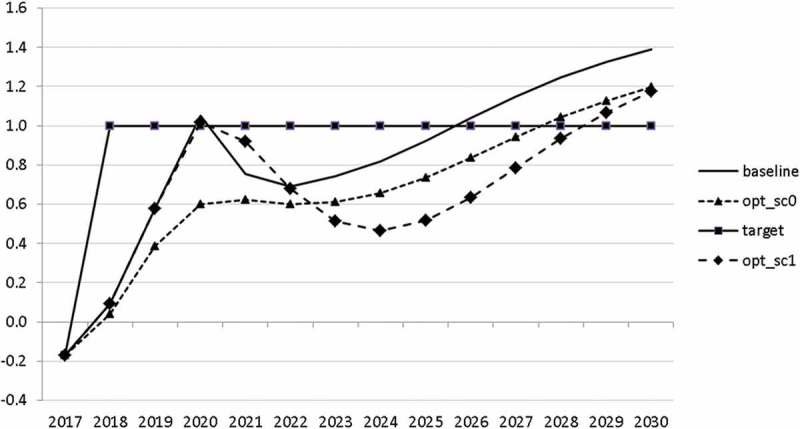
Inflation rate (*INFL*), %.

**Figure 29.**
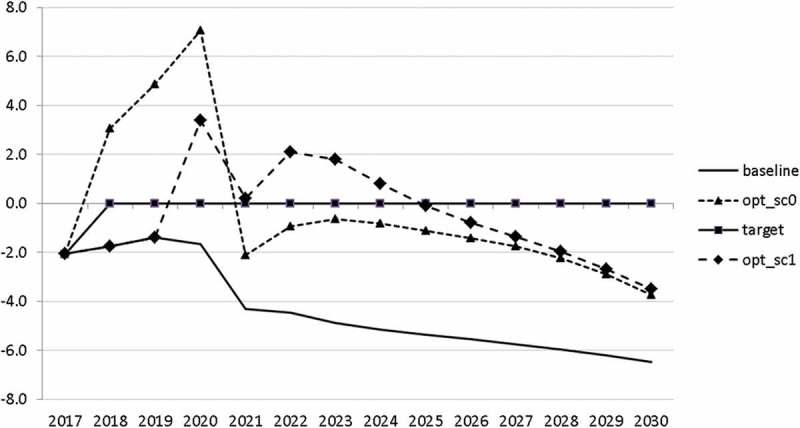
Budget balance ratio to GDP (*BALANCEGDP*), % of GDP.

**Figure 30.**
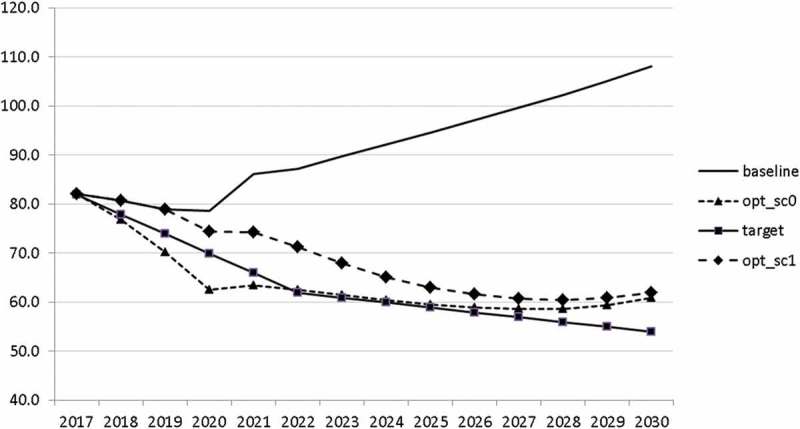
Public debt (*DEBTGDP*), % of GDP.

The other state variables behave in a parallel way to GDP; the unemployment rate is higher (Figure 27) and the inflation rate is lower (Figure 28) than in the absence of the shock. In the uncontrolled projection the government is confronted with high budget deficits (Figure 29) and quite sharply rising public debt (Figure 30), arriving at the level of 110% of GDP in 2030. This explains why a more restrictive fiscal policy is calculated to be optimal in the crisis scenario. This is the only way to have government finances under control, with relatively small negative side effects on output and employment.

## 7. Conclusions

In this article, we used the macroeconometric model SLOPOL10 to calculate simulations of the development of the Slovenian economy until 2030. Starting from the present favourable prospects of the European economies, the forecast is very optimistic but it can nevertheless be improved by optimal fiscal policies as calculated using the OPTCON2 algorithm. If a negative shock to world trade of a size comparable to the Great Recession occurs, it will entail a decline in GDP and a slow recovery, as has to be expected. In this case, optimal fiscal policies should not act in an expansionary way as the effectiveness of fiscal policy with respect to output and employment is rather limited in a small open economy like Slovenia. Instead, the goal of budget consolidation will call for a more restrictive fiscal policy, at least if (as assumed) the shock is temporary. Thus typical Keynesian fiscal policy advice is not optimal even in a model with strong Keynesian features like the one used here.

The main contributions of the article are the following. We present the first simulation of different scenarios for the movements of Slovenian macroeconomic aggregates based on the information before and during the economic crisis. We present one of the rare analyses of fiscal policies simulations for a former Yugoslav country, which could be of interest to other countries that intend to become members of the European Union. In addition, our analysis provides information on macroeconomic policies in situations of a recession that counter the classical Keynesian arguments and present important information for the macroeconomics of a small open economy without autonomous monetary policy.

Our analysis opens up important questions which could be extended and explored in future research. On the one hand, the model could be extended in macroeconometric terms to allow for stochastic components, mixed frequency data (which would allow for including additional control variables) and non-equilibrium (e.g. agent-based) approaches. Also, the findings could be put in the macroeconomic context of other countries of comparable macroeconomic characteristics. The finding that the typical expansionary Keynesian fiscal policy advice is not optimal even in a model with strong Keynesian features should be tested and explored on other macroeconomic situations and contexts, verifying it in similar situations for other countries and other types of crisis contexts (possibly, supply-side based). Finally, the time horizon could be extended to allow for long-term projections, with the help of other forecasting modelling approaches.

## Note

1. We used the EUROPOP population projection from the Eurostat database; this projection is also available from the Statistical Office of the Republic of Slovenia.
